# Supplier Selection and Order Allocation under a Carbon Emission Trading Scheme: A Case Study from China

**DOI:** 10.3390/ijerph17010111

**Published:** 2019-12-22

**Authors:** Chen Wang, Qingyan Yang, Shufen Dai

**Affiliations:** Donlinks School of Economics and Management, University of Science and Technology Beijing, Beijing 100083, China; chenwang@ustb.edu.cn (C.W.);

**Keywords:** supplier selection, order allocation, multiple-criteria decision-making, integer programming, carbon emission trading scheme

## Abstract

In implementing carbon emission trading schemes (ETSs), the cost of carbon embedded in raw materials further complicates supplier selection and order allocation. Firms have to make decisions by comprehensively considering the cost and the important intangible performance of suppliers. This paper uses an analytic network process–integer programming (ANP–IP) model based on a multiple-criteria decision-making (MCDM) approach to solve the above issues by first evaluating and then optimizing them. The carbon embedded in components, which can be used to reflect the carbon competitiveness of a supplier, is integrated into the ANP–IP model. In addition, an international large-scale electronic equipment manufacturer in China is used to validate the model. Different scenarios involving different carbon prices are designed to analyze whether China’s current ETS drives firms to choose more low-carbon suppliers. The results show that current carbon constraints are not stringent enough to drive firms to select low-carbon suppliers. A more stringent ETS with a higher carbon price could facilitate the creation of a low-carbon supply chain. The analysis of the firm’s total cost and of the total cost composition indicates that the impact of a more stringent ETS on the firm results mainly from indirect costs instead of direct costs. The indirect cost is caused by the suppliers’ transfer of part of the low-carbon investment in the product, and arises from buying carbon permits with high carbon prices. Implications revealed by the model analysis are discussed to provide guidance to suppliers regarding the balance between soft competitiveness and low-carbon production capability and to provide guidance to the firm on how to cooperate with suppliers to achieve a mutually beneficial situation.

## 1. Introduction

Greenhouse gas emissions (GHGs) from anthropogenic activities are the main contributors to climate change according to the fourth report of the International Panel on Climate Change [1]. Many countries have actively explored ways to reduce GHGs, and carbon emission trading schemes (ETSs) have become one of the most popular ways to meet CO_2_ mitigation targets because of the flexibility and cost effectiveness that ETSs provide [2,3]. China has built seven ETS city pilots since 2012, and the country also built a national carbon market, which was completed by the end of 2017. ETSs provide enterprises with an economic way to reduce GHGs. However, the carbon constraints allocated under an ETS have also compelled firms to adjust their operational strategies, including strategies for low-carbon supplier selection and order allocation.

Supplier selection is an important strategic decision in developing a competitive supply chain because firms often outsource raw materials, components, or services to suppliers to enhance the core competitiveness of the firm [4]. Moreover, high procurement costs induce firms to select suppliers very cautiously and to establish long-term collaborative relationships with fewer suppliers [5]. Consequently, choosing the appropriate supplier is vital to improve the quality of the final products, enhance business continuity, and maintain the strategic competitiveness of a firm [6]. Traditionally, supplier selection is influenced by various tangible and intangible criteria, such as price, quality, delivery time, and technical capability [7,8]. As the public pays increasingly more attention to the issues of environmental pollution, resource depletion, wealth inequality, and the corporate social responsibility exposed during the operations of firms, managers are required to concentrate on not only the economic effects, but also the environmental and social effects of corporate operations [9,10,11].

However, currently, low-carbon development has put higher demands on supplier selection, especially for firms under an ETS. After the implementation of an ETS, selecting the best supplier becomes a more complex multiple-criteria decision-making (MCDM) issue. In terms of economic criteria, the low-carbon capability of the supplier affects the cost structure of a firm with carbon quota constraints in two ways. On the one hand, from the perspective of the whole product life cycle, the carbon emissions generated by suppliers during raw material or component production—these emissions are called embedded carbon—are an important part of the GHGs emitted by firms. Raw materials embedded with a higher carbon content may generate a cost for extra carbon emissions. On the other hand, low-carbon raw material often has a higher price, which increases the procurement costs of the firm. Therefore, considering carbon price fluctuations, operational decisions regarding supplier selection and order allocation should be adjusted to correspondingly achieve the optimal performance of economic indicators [12]. More importantly, economic indicators are not the only criteria for low-carbon supplier selection. A supplier’s performance in terms of traditional indicators, such as quality and delivery, cannot be ignored under an ETS. 

Therefore, low-carbon supplier selection under an ETS presents two challenges. The first challenge is to determine how to combine both tangible economic criteria and intangible traditional criteria. The second challenge is to determine how to minimize total cost by balancing the carbon cost of the embedded carbon in raw materials and the procurement cost caused by the high price of low-carbon materials. Concerning the establishment of China’s national carbon market, three issues need to be addressed:

(1) How can manufacturing firms with carbon constraints choose suppliers and allocate orders among them while considering the costs of embedded carbon?
(2) Have the current ETSs in China driven Chinese manufacturing firms to choose low-carbon suppliers?
(3) How will a more stringent ETS impact supplier selection strategies?

To cope with the abovementioned challenges and to answer the above three questions, this paper uses an analytic network process–integer programming (ANP–IP) model to solve the issues of low-carbon supplier selection and order allocation for a firm constrained by an ETS with multiple manufacturing plants and suppliers. The cost of the carbon embedded in the components provided by suppliers is also considered in the model; the direct and indirect costs generated by the ETS can be analyzed based on the cost of the embedded carbon. An international large-scale electronic equipment manufacturer in China is used to validate the model. In addition, different scenarios are used to analyze the impact of different carbon constraints on the supplier selection strategies of firms.

The rest of the paper is organized as follows. In Section 2, we review related studies. Section 3 introduces the case firm, elaborates on the research framework, and describes the methodology used to determine the solution. In Section 4, the effectiveness of the proposed model is verified by evaluating the case firm. Low-carbon supplier selection and order allocation under different scenarios are evaluated. In each scenario, the soft competitiveness of the supplier is either considered or not considered, and either the current or a more stringent ETS is in effect. In Section 5, we further discuss the cost structure in each scenario. Section 6 provides the conclusion.

## 2. Literature Review

Concerning sustainable development with low-carbon requirements, the aspects considered become more complex when selecting suppliers, and the evaluation methods are required to be more scientific and accurate.

### 2.1. Evaluation Methods for Low-Carbon Supplier Selection and Order Allocation

Many approaches have been proposed to solve the issue of low-carbon supplier selection including data envelopment analysis (DEA), mathematical programming, analytic hierarchy process (AHP), analytic network process (ANP), fuzzy set theory, and hybrids of these approaches. The studies of Ho et al. (2010) and Chai et al. (2013) provided comprehensive overviews of the evaluation methods for supplier selection [13,14]. Each method has its own advantages and disadvantages. 

Some early studies used mathematical programming models, including linear programming (LP), nonlinear programming, and mixed-integer programming (MIP), to solve the supplier selection issue. For example, Hong et al. (2005) proposed a mixed-integer linear programming model to select the best supplier by considering both changes in suppliers’ supply capabilities and customer needs over a specific period. Using a multi-objective programming model, Wadhwa and Ravindran (2007) selected suppliers based on the minimum price, lead time, and rejects [15,16]. Concerning low-carbon development, some of these studies also considered supplier selection issues when optimizing supply chain networks while considering low-carbon concerns. Abdallah et al. (2012), Nouira et al. (2016), and Zouadi et al. (2018) used an MIP model to design a low-carbon supply chain [17,18,19].

The mathematical programming approach accurately measures the relationship between economic benefits and carbon costs. However, supplier selection cannot be measured from only an economic perspective. In particular, concerning sustainable development, many intangible factors that cannot be quantitatively measured, such as long-term partnership, service levels, brands, and the welfare of employees, must be considered. Thus, in order to consider qualitative factors besides quantitative ones, supplier selection should be treated as a multiple-criteria decision-making (MCDM) problem to obtain a comprehensive solution. The corresponding MCDM methods need to be employed to choose a preferred option, categorize the alternatives, and rank them with a preference-based approach [20].

To select the best supplier by using a much more comprehensive perspective, the AHP and ANP have been widely used by scholars. Scholars combine the AHP or the ANP with other theories or models to improve decision-making accuracy. Hashemi et al. (2015) [21] used the ANP and an improved gray relational analysis (GRA) method to weight the criteria and rank green suppliers in the automotive industry. Using the AHP, Torres-Ruiz and Ravindran (2018) [22] assessed the sustainability risk of suppliers by considering economic, environmental, and social risks. By combining the AHP and Technique for Order Preference by Similarity to an Ideal Solution (TOPSIS) methods, Azimifard et al. (2018) [23] and Jain et al. (2018) [24] dealt with the supplier selection issue for Iran’s steel industry and an Indian automobile company, respectively. Govindan et al. (2018) [25] applied ANP to select the best supplier based on corporate social responsibility practices. A modification of Mikhailov’s Fuzzy Preference Programming (FPP) method was proposed by Chamodrakas et al. (2010) [26] in line with Liberatore’s rating scale AHP method to select suppliers in electronic marketplaces. Shaw et al. (2012) [27] used an approach that combined fuzzy-AHP and fuzzy multi-objective linear programming to select suppliers. In this paper, the carbon emission cap set by the ETS is set as a constraint. Büyüközkan and Çifçi (2012) [28] proposed a model which combined the fuzzy ANP, TOPSIS, and Decision-Making Trial and Evaluation Laboratory Model (DEMATEL) to evaluate green suppliers.

AHP- and ANP-based methods are highly capable of dealing with both quantitative and qualitative factors. However, these methods select the best supplier only by ranking the suppliers and cannot determine the order allocation quantity, which is important for firms, especially those under an ETS. Knowing the order quantity of each supplier can help the firm analyze changes in the carbon cost and the procurement cost caused by the implementation of an ETS.

Some scholars attempted to combine the AHP/ANP and mathematical programming models to select suppliers. Xia and Wu (2007) [29] proposed an approach that integrated the AHP and multi-objective MIP to determine the number of suppliers and order quantity. Wu et al. (2009) [30] combined ANP and MIP to optimize supplier selection in the case of a bundling problem. The authors applied a weighted score to calculate the utility of alternatives by using the ANP to approximate the purchasing manager’s subjective evaluation process. The results were used as coefficients of an objective function in MIP to allocate the order quantities among favorable suppliers. Shaw et al. (2012) [27] used an approach that combined fuzzy-AHP and fuzzy multi-objective linear programming to select suppliers. In this paper, the carbon emission cap set by the ETS is set as a constraint. Kumar et al. (2017) [31] applied a fuzzy AHP and a fuzzy multi-objective LP model to allocate orders in a sustainable supply chain. Hamdan and Cheaitou (2017) [6] used the AHP and bi-objective integer programming for supplier selection and the allocation of orders.

### 2.2. Evaluation Criteria for Supplier Selection

#### 2.2.1. Economic Criteria

Whether an AHP/ANP-based or a mathematical programming method is used, economic criteria must be considered in supplier selection. Hashemi et al. (2015) [21] summarized economic criteria mainly in terms of cost, quality, delivery, technology, flexibility, culture, innovativeness, and relationships. In mathematical programming models, the economic criteria are usually reflected as cost minimization or revenue maximization. The cost structure, which is often complex, includes purchasing costs, logistics costs, transaction costs, and inventory costs. Concerning low-carbon development, some studies also consider carbon costs. When using mathematical programming methods, the economic criteria are often described by quantitative data.

The AHP/ANP-based approach makes decision-making more flexible and the economic criteria more diverse, because this approach is used to measure and evaluate qualitative criteria, which constitute most of the criteria. In the current literature, quality criteria often include lot rejection rates concerning product, quality certification, and corrective and preventive capacity [32,33,34]. Delivery criteria often include compliance with due dates, delivery reliability, and waiting times [24,35,36]. Technology criteria are reflected by current manufacturing capacities, research and development (R&D) capacities, etc. [5,21,23,37,38]. 

#### 2.2.2. Environmental Criteria

With the increase in requirements surrounding sustainable and low-carbon development and the improvement in consumers’ environmental awareness, environmental criteria can no longer be neglected in supplier selection. Since some environmental policies use economic measures, such as environmental taxes and ETSs, to regulate firms’ environmental behaviors, environmental indicators (such as carbon costs, resource consumption, and energy consumption) can be quantified to some extent and used in the mathematical programming model. However, some qualitative indicators, such as a supplier’s low-carbon reputation, a supplier’s environmental management system, and environmental training for staff [33], are also considered by scholars and evaluated in the AHP/ANP-based method.

#### 2.2.3. Social Criteria

As one of the three most important dimensions of sustainable development, the social dimension has become increasingly more of a concern for governments and consumers. Many firms, especially large firms, value their social achievements and impact. Therefore, when making decisions, these firms consider the social impact of the performance of their suppliers. In current studies, social criteria often include the public disclosure of social performance, education and training program support, and employee health and safety [5,23,25,38,39].

### 2.3. Contributions of This Paper

According to the above literature review, although there have been many studies regarding supplier selection and order allocation, these studies did not sufficiently consider the carbon embedded in raw materials, which constitutes most of the carbon footprint of the product life cycle. In addition, far too little attention has been paid to the balance between procurement costs and carbon costs in ETSs. This paper uses an ANP–IP model to solve the MCDM issue of supplier selection under an ETS, and seeks to fill these gaps in the literature and contribute to the current research by considering the following three aspects. First, the cost of carbon embedded in components can be used to reflect the carbon competitiveness of a supplier; therefore, the cost of the embedded carbon is integrated into the ANP–IP model. The cost of embedded carbon could help firms deal with the gradually increasing environmental awareness of consumers and tightened ETS constraints. Second, a real-world case is used to validate the model and to analyze whether China’s current ETS affects, as expected, how firms select low-carbon suppliers; this effect has been less frequently discussed. Third, the results could be used to analyze the cost structure of the firm under an ETS by considering the direct and indirect costs caused by the ETS. This case provides inspiration for policy-makers seeking to design policies or regulations to help firms select more low-carbon suppliers and improve the ability of the overall supply chain to achieve low-carbon targets.

## 3. Methodology

**Figure 1:**
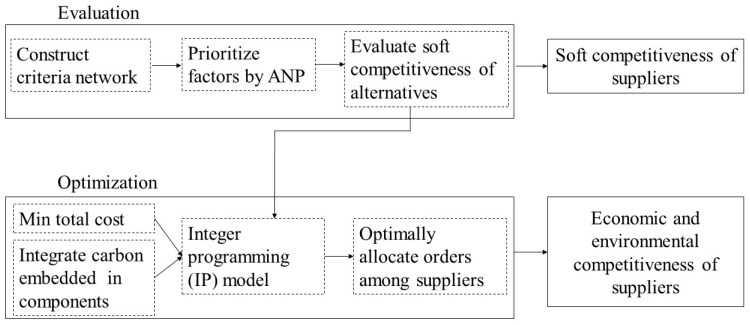
The framework of this paper.

An ANP–IP model considering evaluation and optimization is proposed. The framework of this research is provided in Figure 1. The ANP is first applied to evaluate the soft competitiveness of the suppliers. The evaluation results are then integrated into the IP model as a constraint condition, which is used to optimize the order allocation plan to minimize the total cost. 

### 3.1. Evaluation of the Suppliers’ Soft Competitiveness by Using the ANP

The ANP involves the use of relative measurements with absolute scales for both quantitative and qualitative criteria [40,41,42]. In this paper, the soft competitiveness of a supplier is defined as its comprehensive performance in terms of the criteria that cannot be measured from an economic perspective. The evaluation of the supplier’s soft competitiveness is an MCDM problem involving tangible and intangible criteria, including quality, delivery, service, technology, and social impact. Notably, the low-carbon performance of a supplier is also important for supplier selection; however, this performance cannot be included in the evaluation of the supplier’s soft competitiveness. Under an ETS, buying or selling carbon credits will affect the total cost to the firm, so the low-carbon performance of a supplier has to be excluded from an evaluation of the supplier’s soft competitiveness and integrated into the IP model. The ANP was explained in great detail in Saaty’s book, so the intricacies of the methodology are not introduced in this paper due to space limitations. The ANP is generally divided into the following four steps:

Step 1: Selection of the criteria and construction of the model;
Step 2: Conduction of a pairwise comparison and development of a priority vector with consistency;
Step 3: Construction and limitation of the supermatrix;
Step 4: Evaluation of the alternatives.

#### 3.1.1. Step 1: Selection of the Criteria and Construction of the Model

The ANP consists of criteria, alternatives, and the relationships between them. In our study, the evaluation criteria framework used to evaluate the supplier’s soft competitiveness includes 21 criteria. The criteria were first determined by conducting a detailed analysis of the current literature. We also conducted in-depth interviews with managers in the procurement sector where the case firm operated to make the multiple-criteria indicator framework more practical. The criteria and their influencing relationships to evaluate the supplier’s soft competitiveness are shown in Table 1. In the framework, Q4, Q5, Q6, DS4, and DS5 are recommended by the interviewed managers.

These 21 indicators are divided into 4 clusters: quality, delivery and service, business continuity, and social impact. The quality cluster, which includes indicators Q1–6, reflected the supplier’s performance in terms of product quality. Q1 is the percentage of processed parts that are rejected for a certain number of pieces, which is a direct manifestation of product quality. Q2 indirectly reflects the quality performance of the supplier. Q3 has the potential to improve the quality level. Q5 can affect the rejection rate of a product and influence quality performance. Q6 has the potential to affect the delivery schedule and the performance of the supplier’s after-sales service. The delivery and service cluster reflects the level of the supplier’s on-time delivery and after-sales service, including indicators DS1–5. In this cluster, DS3 affects the capability of handling abnormal quality, the other criteria in the same cluster, and the long-term relationship between the supplier and the manufacturer. DS4 reflects the ability of suppliers to deal with temporary increases and decreases in orders. The business continuity cluster includes indicators BC1–6. BC3 indicates whether the firm is willing to contract with the supplier to cooperate for a long time. Many other indicators affect the long-term relationship between the firm and the supplier. Moreover, once the long-term relationship is formed, it will further affect other aspects of the performance of the supplier. In terms of BC4, suppliers who are flexible in terms of responding to policy changes and are consistent with policy guidance have better business continuity. The social impact cluster, which includes indicators SI1–4, reflects the social requirements of the firm for its suppliers’ performance in terms of sustainable development. 

**Figure 2:**
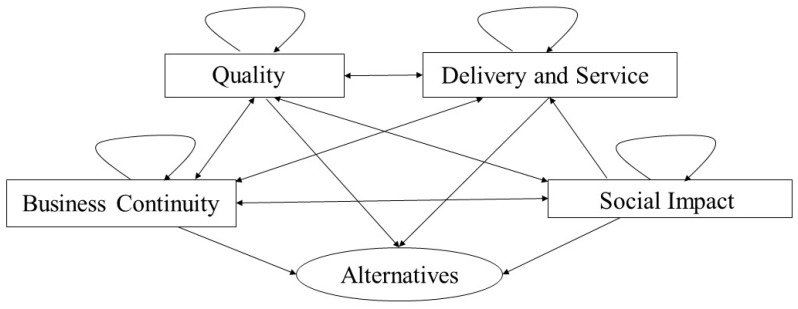
The relationships between the clusters.

The relationships among the clusters are shown in Figure 2. In the ANP model, all suppliers are grouped into an alternative cluster that is affected by all criteria clusters. All the clusters and the complex relationships between them form the ANP model, which is the basis for our subsequent evaluation.

#### 3.1.2. Step 2: Conduction of a Pairwise Comparison and Development of a Priority Vector with Consistency

Once the ANP model was constructed, the criteria were pairwise compared both with and between clusters to form a comparison matrix. In this study, the pairwise comparison includes three types of comparisons, namely, pairwise comparisons of clusters, pairwise comparisons of criteria with regard to their cluster, and pairwise comparisons of the dependencies of the criteria. For the ANP, a 9-point scale indicating how many times an element dominated another is used for comparison. The comparison judgment is translated into numerical values, namely, equally important = 1, moderately more important = 3, much more important = 5, very much more important = 7, and extremely more important = 9.

After the pairwise comparisons have been conducted, each normalized comparison matrix is used to calculate the local priority of every criterion, which will then be used to form a supermatrix. The consistency ratio *CR = CI/RI* was proposed by Saaty to measure the inconsistency of the pairwise comparison, where *RI* was the so-called average consistency index and $CI=\left(\lambda_{max}-n\right)/\left(n-1\right)$ was the consistency index. $\lambda_{max}$ is the principal eigenvalue of the normalized comparison matrix. If the *CR* value is more than 10%, the problem and judgements have to be investigated and revised [40,43].

A reciprocal judgement matrix $A=\left(a_{ij}\right)$ is used to represent judgement. Table 2 shows an example of a pairwise comparison of criteria in the quality cluster with respect to the “long-term relationship”. In the quality cluster, four indicators, namely, Q1–4, affect the long-term relationship. The local priority on the bottom line indicates the importance of these four indicators with respect to the long-term relationship indicator. The inconsistency value is 0.01716, which can be accepted since it does not overcome the threshold established for these pairwise comparison matrices. It should be noted that when multiple decision-makers are involved, the judgement of *m* decision makers is derived by the geometric mean of preference of all of the experts as $a_{ij}={\left(\prod_{k=1}^{m}a_{ijk}\right)}^{1/m}$ [44].

#### 3.1.3. Step 3: Construction and Limitation of the Supermatrix

A supermatrix consisting of all local priority matrices is formed for overall criteria prioritization and alternative ranking. However, this supermatrix is unweighted. The unweighted supermatrix is weighted by multiplying the blocks by the corresponding cluster priority. This weighted supermatrix is eventually used to capture the final influence ranking of the criteria.

#### 3.1.4. Step 4: Evaluating the Alternatives

To evaluate the soft competitiveness of the suppliers, alternatives are also pairwise compared with respect to each criterion. Similar to the process used for criteria ranking, each set of comparison matrices is used to calculate the local rankings of the alternatives; these local rankings are then used to form the supermatrix for the final rankings.

### 3.2. Development of the IP Model to Optimize Order Allocation

With the establishment of ETSs, suppliers have started to adopt more energy-efficient equipment and use low-carbon methods of production to reduce the carbon emissions of their production process. Component suppliers have developed different low-carbon capacities; therefore, these suppliers provide components with different embedded carbon emissions and prices. The ANP model in our study has provided us with the performance and ranking of the three suppliers’ soft competitiveness. The relative score of each supplier is seen as a soft competitiveness index, which is included as one of the constraint conditions of the IP model. This model requires that the soft competitiveness of all of the procured components has to be above average.

The problem lies in deciding which supplier should provide which manufacturing plant components and the quantities of the components that should be provided. The objective is to minimize the total cost. Given that only the relationship between suppliers and manufacturers is considered in this paper, the total cost to be minimized includes the procurement cost for the components, the cost of transporting the components, the cost of producing the final products, and the carbon cost. Under an ETS, the firm is allocated a carbon cap by regulatory agencies. This cap is the total carbon quota constraint for the two manufacturing plants of the firm. If the total carbon emissions of the two plants are higher than the cap, the firm has to buy more carbon permits from the carbon market at the current carbon price to cover the additional emissions. If the total carbon emissions of the two plants are lower than the cap, the firm can sell its additional permits in the carbon market and gain a profit. The total demand for the final products of the two manufacturing plants is assumed to be determined. The variables and parameters used in the IP model are defined in Table 3.

The IP model used for the supplier selection and order allocation under the ETS is calculated as follows:

(1) $$\begin{array}{l} \mathrm{Min}cost=\sum_{s\in S}\sum_{m\in M}q_{sm}^{}(p_{sm}^{}+c_{sm}^{}r_{sm}^{})+\sum_{m\in M}k_{m}^{}q_{m}^{}\\ +p_{c}(\sum_{s\in S}\sum_{m\in M}\rho_{s}^{}q_{sm}^{}+\sum_{s\in S}\sum_{m\in M}\rho_{sm}^{}q_{sm}^{}r_{sm}^{}+\sum_{m\in M}\rho_{m}^{}q_{m}^{}-CO_{2}^{cap})\prime \end{array}$$

s.t.

(2) $$\sum_{s\in S}q_{sm}^{}\geq q_{m}^{},\forall m\in M,$$

(3) $$\sum_{m\in M}q_{m}^{}\geq dem,\forall m\in M,$$

(4) $$q_{m}^{}\leq U_{m}^{},\forall m\in M,$$

(5) $$\frac{\sum_{s\in S}\sum_{m\in M}q_{sm}^{}\lambda_{s}^{}}{\sum_{s\in S}\sum_{m\in M}q_{sm}^{}}\geq 0.33,\forall m\in M,s\in S,$$

(6) $$q_{sm}^{}\geq 0\;\mathrm{and}\;\mathrm{integer},\forall m\in M,s\in S,$$

(7) $$q_{m}^{}\geq 0\;\mathrm{and}\;\mathrm{integer},\forall m\in M.$$

Equation (1) is the objection function to be minimized. The first part of Equation (1) is the procurement cost for the components and the cost of transporting the components from the suppliers to the manufacturers. The second part is the cost of producing the final products, and the third part of Equation (1) is the carbon cost. We define the carbon emissions of the entire process, from purchasing components to production, as Equation (8).

(8) $$CO_{2}^{cur}=\sum_{s\in S}\sum_{m\in M}\rho_{s}^{}q_{sm}^{}+\sum_{s\in S}\sum_{m\in M}\rho_{sm}^{}q_{sm}^{}r_{sm}^{}+\sum_{m\in M}\rho_{m}^{}q_{m}^{}$$

Therefore, the total cost includes the procurement cost for the components, the cost of transporting the components, the cost of producing the final products, and the carbon costs. When $CO_{2}^{cur}>CO_{2}^{cap}$, the carbon cost is positive, thus indicating the firm has to buy carbon permits from the carbon market. However, when $CO_{2}^{cur}<CO_{2}^{cap}$, the carbon cost is negative, thus indicating the firm can sell its extra carbon quota in the carbon market and obtain benefits. Equation (2) ensures that all of the components purchased by the manufacturing plant *m* can meet the production requirements. Equation (3) ensures that the number of final products from all of the manufacturing plants can meet the demand. Equation (4) limits the production of the manufacturing plant *m* so that production cannot exceed the plant’s maximum capacity. Equation (5) ensures that the average soft competitiveness of all of the purchased components is not below the average soft competitiveness of the three suppliers. To achieve cost minimization, the soft competitiveness performance of the suppliers may have been sacrificed to some extent. However, the soft competitiveness of the components cannot be below average. Equations (6)–(7) enforce integrality and nonnegativity on the decision variables.

## 4. Results of the Case Study

### 4.1. Evaluation Results of the Suppliers’ Soft Competitiveness

**Figure 3:**
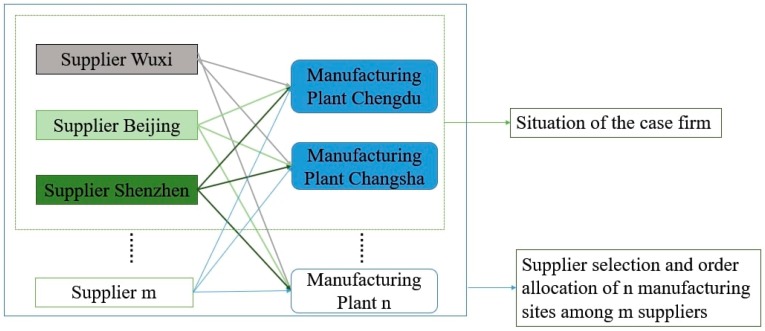
The description of the supplier selection and order allocation issues.

The case firm in this study is an international large-scale electronic equipment manufacturer, and the main products of the company include mobile phones, computers, and electronic watches. In this study, the computer product of the case firm is considered for a specific application of low-carbon supplier selection. Due to the diversity of the raw material and component accessories, to simplify the problem this paper addresses, only suppliers providing the Liquid Crystal Display (LCD) computer screens are considered. The case firm has two manufacturing plants (which are located in Chengdu city and Changsha city) and three LCD suppliers (which are located in Wuxi city, Beijing city, and Shenzhen city) (Figure 3).

In order to evaluate the suppliers’ soft competitiveness, we conducted in-depth interviews with three managers in the procurement department. In addition to helping us improve the criteria framework, each procurement manager was required to compare all of the criteria in pairs and give them a score of 1–9 to reflect the relative importance. Through the interviews, we learned that this electronic equipment manufacturing company considered the low-carbon production capacity of the suppliers when making decisions regarding supplier selection. However, the cost caused by the embedded carbon of the raw materials was not taken into consideration. Based on the ANP described in Section 3.1, the composite scores of the suppliers are synthesized; these are shown in Table 4. Normal values are seen as the soft competitiveness index of each supplier [45]. The results show that from the perspective of soft competitiveness, the supplier in Beijing performs the best, while the Shenzhen supplier performs the worst.

### 4.2. Optimal Results of Supplier Selection and Order Allocation under the ETS

Due to the establishment of China’s unified carbon market in 2017, some suppliers implement low-carbon production. Supplier Shenzhen has put the most effort into low-carbon production, thus resulting in an obvious reduction in the carbon embedded in each unit of product. Supplier Beijing has also implemented a low-carbon production mode, but exhibited less effort than supplier Shenzhen. Supplier Wuxi has made no improvements in low-carbon production. The distances from the suppliers to the manufacturers, the embedded carbon intensities, that is, the embedded carbon emissions per unit of component, and the prices of each supplier are shown in Table 5. The embedded carbon intensity in the product provided by each supplier is estimated by the procurement managers of the firm. Based on Abdallah (2012), the embedded carbon intensities of the most and least low-carbon components are 12.5% lower and 12.5% higher, respectively, than the median.

To reduce the impacts of costs and carbon emissions generated in the assembly process on total cost, we assume that the unit cost of assembling a computer is 1000 Yuan and that the production carbon intensity is 50 kg CO_2_ per unit product. All components are transported by road freight and have a carbon emission coefficient of 0.15 kg CO_2_ per ton per mile. The weight of each screen is approximately 2 kg. The two manufacturing plants are required to meet the order demand of 175,287 computers. The product capacity of each plant does not exceed 100 thousand units. The product capacities of the suppliers are considered to be sufficient to meet the needs of the manufacturers, as they also provide the LCD screen products to computer manufacturers of other brands.

Before the implementation of the ETS, the components offered by the three suppliers had the same embedded carbon intensities and prices. Changes in the decision regarding supplier selection and order allocation are affected by the decision to consider both cost and soft competitiveness. To highlight these changes, Scenario 1 and Scenario 2 are designed to solve these two respective problems for the model.

Scenario 1: Only cost is considered before ETS implementation;

Scenario 2: Both cost and soft competitiveness are considered before ETS implementation.

**Figure 4:**
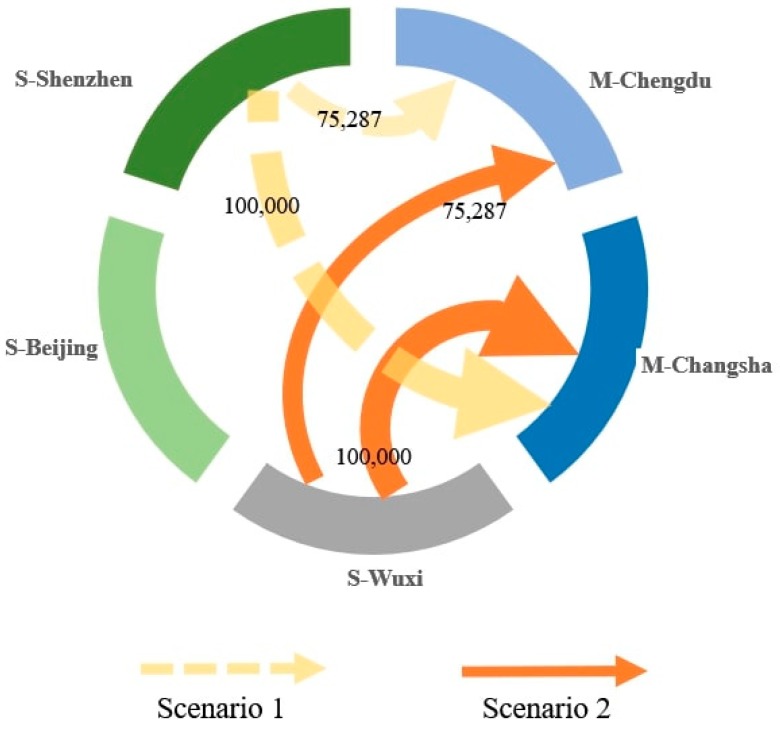
The solutions for Scenarios 1 and 2.

As illustrated in Figure 4, the optimal solutions for the two scenarios are different. In Scenario 1, the Shenzhen supplier provides 75,287 and 100,000 units of LCD screens to the Chengdu and Changsha manufacturing plants, respectively. With the comprehensive consideration of cost and soft competitiveness, the optimal solution in Scenario 2 changes to select the Wuxi supplier, which offers 75,287 and 100,000 units to the Chengdu and Changsha manufacturing plants, respectively.

No carbon cost is generated in either Scenario. The LCD screens offered by the three suppliers have the same price, thus, when considering supplier selection only from the perspective of cost, the only difference between the suppliers is the transportation cost. In Scenario 1, the optimal decision is to choose the Shenzhen supplier, which is closest to the two plants. However, when considering soft competitiveness, the Wuxi supplier is selected instead because the Shenzhen supplier performs worse than the other two suppliers in terms of soft competitiveness and cannot meet the requirements of the firm. Supplier Wuxi has a relatively higher soft competitiveness level and is closer to the two plants.

The carbon emissions before the implementation of the ETS is an important benchmark for the allocation of free carbon quotas. Therefore, we calculate the carbon emissions of the firm in Scenario 1 and Scenario 2 to be 48.61 and 48.77 thousand tons of carbon emissions, respectively. The difference in carbon emissions between the two scenarios is mainly due to the change in transportation distance after changing the supplier selection and order allocation plan. Under the current ETS in China, the free quota allocated to a firm generally accounts for 75–90% of its historical annual carbon emissions. Therefore, to further analyze the impact of ETS implementation on supplier selection and order allocation, it is assumed that the total amount of the free quota allocated to the firm is 40 thousand tons. In China’s actual carbon market, the average carbon price is set to the equivalent of 30 Yuan/ton CO_2_. Scenarios 3 and 4 are designed to analyze the impact of soft competitiveness.

Scenario 3: Only cost is considered under the current ETS;

Scenario 4: Both cost and soft competitiveness are considered under the current ETS. 

**Figure 5:**
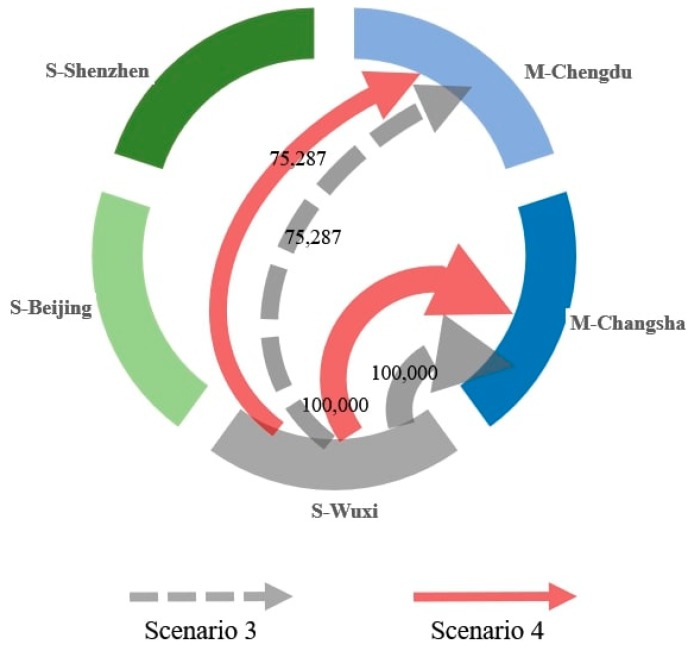
The solutions for Scenarios 3 and 4.

The solutions to Scenarios 3 and 4 are illustrated in Figure 5. The results indicate that the optimal solutions for the two scenarios are the same. In scenario 3, the Wuxi supplier provides 75,287 and 100,000 units of product to the Chengdu and Changsha plants, respectively. When incorporating soft competitiveness, the decision remains the same because the Wuxi supplier has a relatively higher soft competitiveness level.

However, comparing Scenario 3 with Scenario 1 shows that after ETS implementation, the firm chooses the Wuxi supplier, which has the worst low-carbon performance. This indicates that the current carbon quota constraints have not driven the firm to select the low-carbon suppliers, Beijing and Shenzhen. In contrast, because these two suppliers have higher-priced products, the firm prefers the Wuxi supplier due to the lower product price. That is, in Scenario 3, the increase in the procurement cost is higher than the carbon cost. After balancing the carbon cost and procurement cost, the Wuxi supplier, whose product has the lowest price and the highest embedded carbon intensity, becomes the optimal selection.

To achieve the carbon emission reduction target, carbon quota constraints will be gradually strengthened in the future, thereby leading to an increase in the carbon price. The National Development and Reform Commission of China stated that 200–300 Yuan per ton is the ideal carbon price. Thus, Scenarios 5 and 6 are designed to analyze whether more stringent carbon quota constraints will drive firms to select low-carbon suppliers. The carbon price is set to 300 Yuan per ton.

Scenario 5: Only cost is considered under a more stringent ETS;

Scenario 6: Both cost and soft competitiveness are considered under a more stringent ETS.

**Figure 6:**
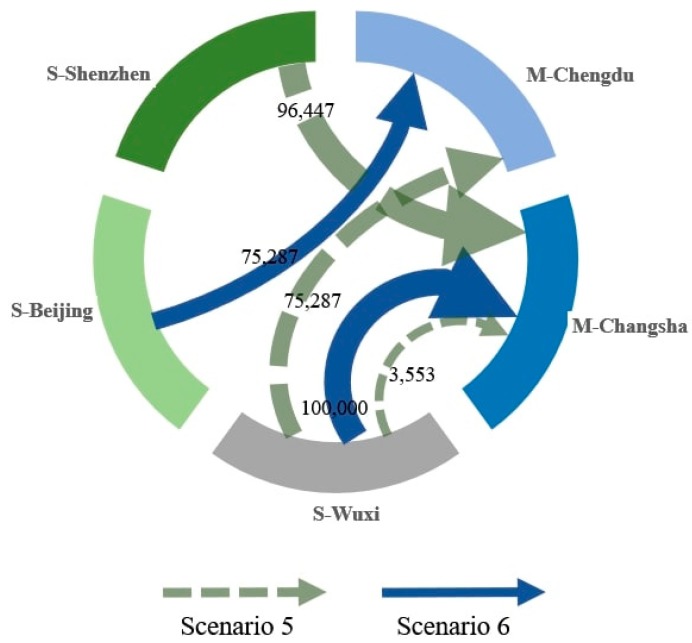
The solutions for Scenarios 5 and 6.

The solutions for Scenarios 5 and 6 are illustrated in Figure 6. Under scenario 5, the optimal decision is to purchase 96,447 LCD screens from the Shenzhen supplier for the Changsha plant, 75,287 units from the Wuxi supplier for the Chengdu plant, and 3553 units from the Wuxi supplier for the Changsha plant. Compared to Scenario 3, where the carbon price is 30 Yuan per ton, the optimal decision has clearly changed.

Under more stringent carbon constraints, some components are purchased from the most low-carbon supplier, which is the Shenzhen supplier, thus indicating that after balancing the carbon cost with other costs, the firm starts to consider the low-carbon production capacity of the supplier. In Scenario 6, the Shenzhen supplier is no longer considered because of its poor performance in terms of soft competitiveness. Meanwhile, although the distance from the Beijing supplier to the two plants is the longest, the relatively stronger low-carbon capacity and the highest level of soft competitiveness of the Beijing supplier help the supplier gain an order of 75,287 units for the Chengdu plant.

## 5. Analysis and Discussion of the Cost Structure

**Figure 7:**
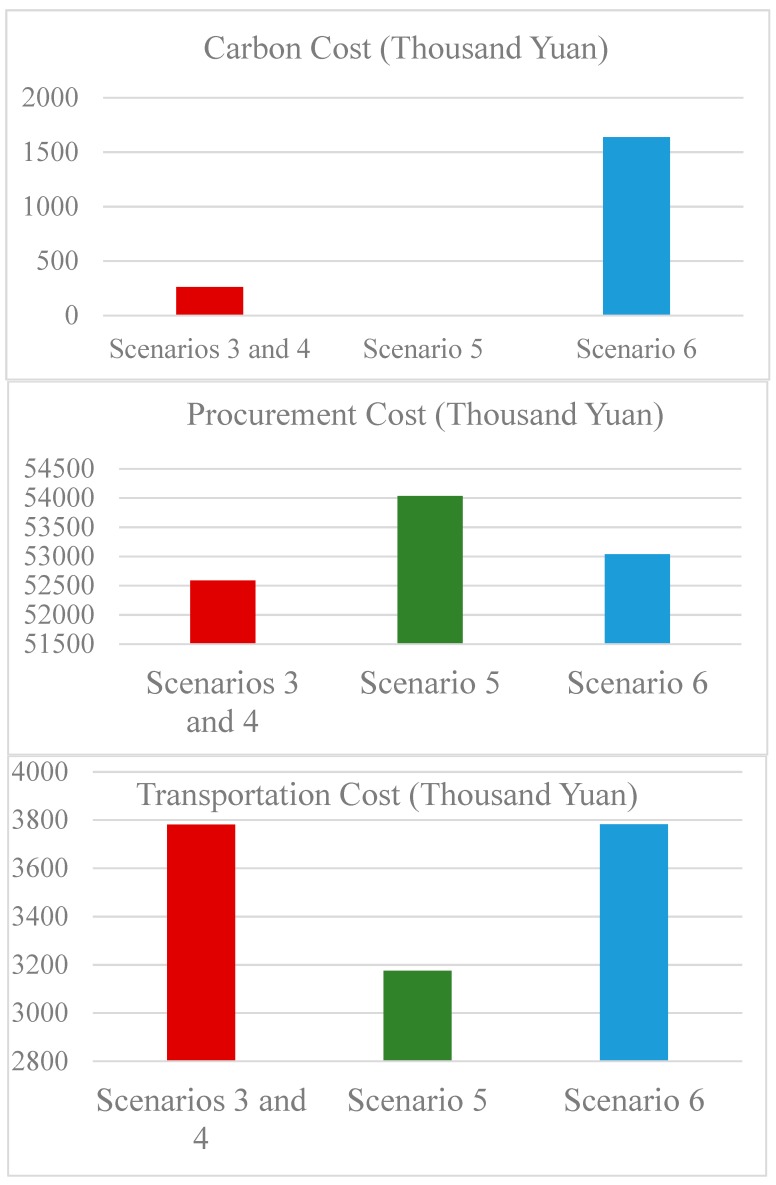
Comparison of the cost structures of different scenarios.

By applying the above ANP-IP model to the case firm under China’s ETS, it can be seen that whether a decision regarding supplier selection and order allocation is optimal is directly affected by comprehensive consideration of the indicators and the stringency of the carbon quota constraints. The cost structures of the firm in different scenarios are further analyzed in this paper (Figure 7). Only the carbon cost, the procurement cost of the LCD screens, and the transportation cost are included in our analysis because the production costs in the different scenarios are the same. The carbon cost depends on the total carbon and the carbon price, so the carbon cost can be seen as a direct cost pressure caused by the ETS on the firm. The procurement cost of each scenario is different because after the implementation of the ETS, the suppliers transfer a part of the low-carbon investment to their LCD products. Therefore, the increase in the procurement cost can be seen as an indirect cost pressure caused by the ETS.

The difference between Scenario 3 and Scenario 5 is caused by the substantial increase in carbon prices. In Scenario 3, all orders are allocated to the least low-carbon supplier (Wuxi), while in Scenario 5, some of the orders are allocated to the most low-carbon supplier (Shenzhen). The different order allocations in these scenarios indicate that the more stringent ETSs with higher carbon prices will affect the optimal decision concerning supplier selection and order allocation. Comparing the cost structures in the two scenarios shows that no carbon cost is generated in Scenario 5, therefore, the increase in the carbon price does not cause a large increase in the carbon cost. Meanwhile, the transportation distance in Scenario 5 is shorter, resulting in a much smaller transportation cost. The only increased cost in Scenario 5 results from the improved procurement price of the LCD screen, that is, the impact of the more stringent ETS on the firm does not result from the direct cost pressure caused by the high carbon price, but from the indirect cost pressure caused by the suppliers’ transfer of a part of the low-carbon investment to the product. Meanwhile, if the low-carbon technologies and investment of supplier Shenzhen achieve scale, the production and management costs will be reduced, thus leading to a lower price of the supplier’s LCD screen. In terms of cost, the Shenzhen supplier will have a greater competitive advantage in the industry.

In both Scenario 4 and Scenario 6, both the cost and the soft competitiveness of the alternatives are comprehensively considered in the selection of suppliers and the allocation of orders. In Scenario 4, the firm chooses the least low-carbon supplier (Wuxi) for all of the LCD screens. However, in Scenario 6, which is affected by the more stringent ETS with a higher carbon price, the firm allocates some of the orders to the relatively more low-carbon supplier (Beijing). The transportation distances in the two scenarios are almost the same, thus leading to almost identical transportation costs. Compared with the carbon cost in Scenario 4, the carbon cost in Scenario 6 is significantly higher, that is, when considering the suppliers’ soft competitiveness, the strict ETS will greatly increase the direct cost pressure on the firm. An indirect cost pressure of the ETS is also generated, but the extent is relatively small. The Wuxi supplier has stronger soft competitiveness, but the supplier has to be abandoned by the firm under the stricter ETS because its embedded carbon intensity of the components supplied by the supplier is too high. Therefore, the supplier with strong soft competitiveness should engage in low-carbon production as soon as possible to gain an even greater competitive advantage after the implementation of the ETS.

The difference between Scenario 5 and Scenario 6 is the consideration of the supplier’s soft competitiveness for supplier selection. In Scenario 5, some of the LCD screens are purchased from the most low-carbon supplier, i.e., Shenzhen. Thus, even if the carbon price is very high at this time, no carbon cost is generated, that is, the stringent ETS does not exert a direct cost pressure on the enterprises. However, in Scenario 6, the most low-carbon supplier (Shenzhen) is replaced by the other two suppliers because of its poor performance in terms of soft competitiveness. Although the procurement cost decreases to some extent, the carbon cost and transportation cost increase significantly, thus resulting in an increase in the total cost. Therefore, the suppliers that have already paid attention to low-carbon production should consider the coordinated development of soft competitiveness and low-carbon production capacity to obtain a strategic competitive position among industrial competitors. In addition, if the firm can build long-term relationships with low-carbon suppliers and cooperates to improve the suppliers’ capabilities and performances in all aspects, the firm can achieve a mutually beneficial result.

## 6. Conclusions

The ETS has become one of the most popular ways to reduce GHGs. Under the ETS, the selection of the best supplier becomes a more complex MCDM issue and presents two key challenges. The first challenge regards the combination of both tangible and intangible indicators involved in this issue. The second challenge revolves around the balance the cost of carbon embedded in raw materials and other costs, such as procurement cost.

To solve the problems of supplier selection and order allocation under an ETS, an analytic network process–integer programming (ANP–IP) model, which first evaluates and then optimizes the problem, is proposed. The carbon embedded in the components offered by the suppliers is also considered. We first construct an evaluation framework for each supplier’s soft competitiveness, which includes multiple important criteria that needs to be considered in terms of quality, delivery and service, business continuity, and social impact. The ANP is applied to evaluate each firm’s performance and to calculate a soft competitiveness index for each supplier. This soft competitiveness index is integrated into the constraint conditions of the IP model to determine the optimal supplier combination and the order allocation plan in order to minimize the total cost. By using this model, multiple intangible criteria for the selection of suppliers is comprehensively considered, and order allocation among suppliers is determined to minimize the total cost.

A real-world-based case of an international large-scale electronic equipment manufacturer with two plants that manufacture computer products and three LCD screen suppliers located in China is used to validate the model. This paper provides an approach for the decision-maker of the case firm to select suppliers under an ETS, whereby the case firm can consider the carbon embedded in the components to minimize the total cost without losing traditional soft competitiveness performance when allocating the order. The results also clearly demonstrate the impact of changes in the supplier selection strategy on the firm’s total cost and total cost composition under ETS scenarios with different constraint strengths. Correspondingly, we show how suppliers can balance soft competitiveness and low-carbon production capability to maintain their competitive position. We also show how the case firm can cooperate with its suppliers to achieve a mutually beneficial situation.

The current carbon quota constraints are not stringent enough to drive the case firm to select a low-carbon supplier. When the price of the products increases, the firm prefers the least low-carbon supplier because the supplier has the lowest price. However, a more stringent ETS with a higher carbon price will affect the optimal decision concerning supplier selection and order allocation. The impact of the more stringent ETS on the firm does not result from the direct cost pressure caused by the high carbon price but from the indirect cost pressure caused by the suppliers’ transfer of part of the low-carbon investment in the product.

Suppliers with strong soft competitiveness should engage in low-carbon production as soon as possible to maintain their competitive position after the implementation of an ETS. As soon as suppliers can achieve a relatively strong low-carbon capability, they should try to make investments to achieve scale and to reduce the price of their low-carbon products, which can help suppliers achieve an even greater competitive advantage. Moreover, soft competitiveness and low-carbon production capacities have to be developed in a coordinated fashion. Finally, if firms build long-term relationships with low-carbon suppliers and cooperate to improve the suppliers’ capabilities and performance, firms can achieve results that are beneficial for both parties.

## Figures and Tables

**Table 1:** The multiple criteria framework used to evaluate the suppliers’ soft competitiveness.

Criteria (Abbreviation)	Definition	Influencing Factors
Lot rejection rate of the product (Q1)	The percentage of processed parts that are rejected for a certain number of pieces.	Q5, DS1, BC1, BC2, BC6
Quality management system and certificates (Q2)	A good and complete quality management system and whether it is certified by an authority.	Q4, BC3, BC5, BC6
Capability of handling abnormal quality (Q3)	A systematic way of handling (potential) negative feedback or any factors that cannot meet customers’ expectations.	Q1, Q4, DS3, BC1, BC5, BC6
Traceability system (Q4)	A complete set of measures that can be used to identify specific aspects that cause quality problems.	Q2, Q5, BC1, BC3
Inspection technology and capacity (Q5)	Advanced equipment and scientific inspection methods are applied to inspect product quality during production.	Q4, DS1, BC1, BC3,
Containment action (Q6)	The capability of the firm to immediately respond to any quality issue.	Q1, Q2, Q3, Q4, Q5, DS1, DS2, BC4, SI1
Delivery schedule (DS1)	Whether the supplier can deliver the order quantity on time.	Q1, Q3, Q4, Q5, Q6, DS3, DS4, BC1, BC2, BC3, BC4, BC5, BC6
After-sales service (DS2)	The capability, attitude, and technical support level of the supplier for follow-up service after the order is complete.	Q1, Q2, Q3, Q4, Q5, Q6, DS1, DS3, BC1, BC3, BC4, BC5, BC6, SI1
Response to specific requests (DS3)	A complete service management system to deal with occasional or unconventional requirements of customers.	Q2, Q3, Q4, Q5, DS1, DS2, DS5, BC1, BC2, BC3, BC5, BC6
Response to the MPS (master production schedule) variance (DS4)	The flexibility of production and service, reflecting the ability of suppliers to deal with temporary increases or decreases in orders.	Q1, Q2, Q3, Q4, Q5, DS1, DS3, BC1, BC3, BC5, BC6
Capacity of new product initiation (DS5)	A complete service system and corresponding responsible people to address the order of a new product.	Q2, Q4, Q5, DS3, BC1, BC2, BC3, BC5, BC6
Technology level (BC1)	The current production technology level of a supplier.	Q5, BC2, BC6
Capacity of R&D (BC2)	Whether the supplier has sufficient capacity to maintain or even improve its technology level.	Q2, Q5, BC1, BC3, BC6
Long-term relationship (BC3)	Whether the firm is willing to contract with the supplier to cooperate for a long time.	Q1, Q2, Q3, Q4, DS1, DS2, DS3, DS4, DS5, BC1, BC2, BC4, BC5, BC6, SI1
Response to government policies and regulations (BC4)	The sensitivity of the supplier to relevant policies and regulations.	Q4, BC1, BC2, BC5, BC6
Clear and reasonable organizational structure (BC5)	There are neither overlapping responsibilities nor unclaimed responsibilities between sectors.	Q2, BC6
Learning and development opportunities for employees (BC6)	A complete employee training and education system, clear standards, and fair opportunities for promotion.	Q2, BC5
Public disclosure of environmental and social performance (SI1)	Regular disclosures of the firm’s efforts in terms of social welfare improvement and environmental protection.	BC1, BC4, BC5, SI2, SI3, SI4
Support for education and job training programs (SI2)	The capability of providing sufficient job opportunities. The firm establishes scholarships and provides visiting or training programs for members of society.	Q2, BC5
Employee health and safety (SI3)	The reputation in society in terms of providing a good working environment. The firm promises to protect the health and safety of its employees.	BC1, BC5, SI1, SI4
Compliance with labor laws (SI4)	Whether the supplier has violated labor laws, such as by employing child labor.	BC5, SI1, SI3

**Table 2:** Pairwise comparisons of the criteria in the quality cluster with respect to the long-term relationship.

	Q1	Q2	Q3	Q4
Q1	1	1	3	2
Q2	1	1	2	2
Q3	1/3	1/2	1	1/2
Q4	1/2	1/2	2	1
Local priorities	0.3564	0.3257	0.1243	0.1936

Inconsistency: 0.01716.

**Table 3:** Symbols and definitions of the parameters and variables.

Type	Symbol	Definition
*Sets*	*S*	Set of the suppliers, indexed by *s*
	*M*	Set of the manufacturers, indexed by *m*
*Parameters*	*ρ_s_*	Carbon emissions per unit of component provided by supplier *s*
	*ρ_sm_*	Carbon emission factor for the transportation from supplier *s* to manufacturer *m* in tons per mile
	*ρ_m_*	Carbon emissions per unit of product in manufacturing plant *m*
	*p_sm_*	Price of the component provided by suppler *s* to manufacturing plant *m*
	*k_m_*	Unit production cost of the manufacturing plant *m*
	*c_sm_*	Unit transportation cost from supplier *s* to manufacturing plant *m*
	*r_sm_*	Distance from supplier *s* to manufacturing plant *m*
	*λ_s_*	Soft competitiveness index of supplier *s*
	*p_c_*	Carbon price in the carbon market
	*CO*_2_ *^cap^*	Free carbon quotas allocated to the firm
	*CO*_2_ *^cur^*	Actual carbon emissions
	*U_m_*	Maximum production capacity of manufacturing plant *m*
	*dem*	Order demand for all the manufacturing plants of the firm
*Decision variables*	*q_sm_*	Number of components provided by supplier *s* to manufacturing plant *m*
	*q_m_*	Number of final products made by manufacturing plant *m*

**Table 4:** Alternative rankings of soft competitiveness.

Suppliers	Raw Score	Ideal	Normal	Ranking
Supplier Wuxi	0.0624	0.9886	0.3755	2
Supplier Beijing	0.0632	1	0.3798	1
Supplier Shenzhen	0.0407	0.6645	0.2448	3

**Table 5:** The values of some relative parameters.

	Supplier Wuxi	Supplier Beijing	Supplier Shenzhen
Distance to manufacturer Chengdu (km)	1548	1518	1343
Distance to manufacturer Changsha (km)	823	1609	358
Embedded carbon intensity (kg CO_2_ per unit product)	225	181	135
Price (Yuan per unit product)	300	306	315
